# Retraction: Genetic Polymorphism of Glucokinase on the Risk of Type 2 Diabetes and Impaired Glucose Regulation: Evidence Based on 298, 468 Subjects

**DOI:** 10.1371/journal.pone.0261919

**Published:** 2021-12-22

**Authors:** The PLOS ONE Editors

Following the publication of this article [1], the editorial team has become aware of a highly similar manuscript that was previously submitted for consideration in *PLOS ONE* and subsequently withdrawn by the authors. Despite high similarity between the two bodies of work, the authors and affiliations listed on the withdrawn submission are different from the authors and affiliations listed on the published article.

The authors have not responded to communications regarding the similarity between the submissions and the authorship concerns. It remains unclear who conducted the research described in this article [1] and whether the listed authors met authorship criteria of the *PLOS ONE* policy.

In light of the unresolved concerns the *PLOS ONE* Editors retract this article.

The authors either did not respond directly or could not be reached.
